# Dicer-mediated miR-200b expression contributes to cell migratory/invasive abilities and cancer stem cells properties of breast cancer cells

**DOI:** 10.18632/aging.204205

**Published:** 2022-08-08

**Authors:** Tung-Wei Hsu, Hsin-An Chen,, Po-Hsiang Liao, Yen-Hao Su, Ching-Feng Chiu, Chih-Yang Huang, Yu-Jung Lin, Chih-Chiang Hung, Ming-Hsin Yeh, Shian-Ying Sung, Chih-Ming Su

**Affiliations:** 1 Graduate Institute of Medical Sciences, College of Medicine, Taipei Medical University, Taipei 11031, Taiwan; 2 Division of General Surgery, Department of Surgery, Shuang Ho Hospital, Taipei Medical University, New Taipei City 23561, Taiwan; 3 Graduate Institute of Clinical Medicine, College of Medicine, Taipei Medical University, Taipei 11031, Taiwan; 4 Department of General Surgery, School of Medicine, College of Medicine, Taipei Medical University, Taipei 11031, Taiwan; 5 TMU Research Center of Cancer Translational Medicine, Taipei Medical University, Taipei 11031, Taiwan; 6 Nutrition Research Center, Taipei Medical University Hospital, Taipei 11031, Taiwan; 7 Graduate Institute of Metabolism and Obesity Sciences, College of Nutrition, Taipei Medical University, Taipei 11031, Taiwan; 8 Cardiovascular and Mitochondrial Related Disease Research Center, Hualien Tzu Chi Hospital, Buddhist Tzu Chi Medical Foundation, Hualien 97002, Taiwan; 9 Center of General Education, Buddhist Tzu Chi Medical Foundation, Tzu Chi University of Science and Technology, Hualien 97002, Taiwan; 10 Department of Medical Research, China Medical University Hospital, China Medical University, Taichung 404, Taiwan; 11 Graduate Institute of Biomedical Sciences, China Medical University, Taichung 404, Taiwan; 12 Division of Breast Surgery, Department of Surgery, Taichung Veterans General Hospital, Taichung 40705, Taiwan; 13 Department of Applied Cosmetology, College of Human Science and Social Innovation, Hungkuang University, Taichung 433, Taiwan; 14 Department of Surgery, Chung Shan Medical University Hospital, Taichung 40201, Taiwan; 15 Institute of Medicine, School of Medicine, Chung Shan Medical University, Taichung 40201, Taiwan; 16 International Ph.D. Program for Translational Science, College of Medical Science and Technology, Taipei Medical University, Taipei 11031, Taiwan; 17 Office of Human Research, Taipei Medical University, Taipei 11031, Taiwan; 18 TMU-Research Center of Urology and Kidney, Taipei Medical University, Taipei 11031, Taiwan; 19 Clinical Research Center, Taipei Medical University Hospital, Taipei 11031, Taiwan; 20 Graduate Institute of Clinical Medicine, School of Medicine, College of Medicine, Taipei Medical University, Taipei 11031, Taiwan

**Keywords:** dicer, cancer stem cells properties, migration, invasion, miR-200b

## Abstract

Distant metastasis is the leading cause of death in patients with breast cancer. Despite considerable treatment advances, the clinical outcomes of patients with metastatic breast cancer remain poor. CSCs can self-renew, enhancing cancer progression and metastasis. Dicer, a microRNA (miRNA) processing–related enzyme, is required for miRNA maturation. Imbalanced Dicer expression may be pivotal in cancer progression. However, whether and how Dicer affects the stemness of metastatic breast cancer cells remains unclear. Here, we hypothesized that Dicer regulates the migration, invasion, and stemness of breast cancer cells. We established highly invasive cell lines (MCF-7/I-3 and MDA-MB-231/I-3) and observed that Dicer expression was conspicuously lower in the highly invasive cells than in the parental cells. The silencing of Dicer significantly enhanced the cell migratory/invasive abilities and CSCs properties of the breast cancer cells. Conversely, the overexpression of Dicer in the highly invasive cells reduced their migration, invasion, and CSCs properties. Our bioinformatics analyses demonstrated that low Dicer levels were correlated with increased breast cancer risk. Suppression of Dicer inhibited miR-200b expression, whereas miR-200b suppression recovered Dicer knockdown–induced migration, invasion, and cancer stem cells (CSCs) properties of the breast cancer cells. Thus, our findings reveal that Dicer is a crucial regulator of the migration, invasion, and CSCs properties of breast cancer cells and is significantly associated with poor survival in patients with breast cancer.

## INTRODUCTION

Breast cancer is one of the most common malignant neoplasms in women and remains a leading cause of morbidity worldwide [1]. In the United States, the prevalence of breast cancer is one per eight individuals, and a woman is diagnosed as having breast cancer every 2 min [2, 3]. Although breast cancer treatment has considerably advanced over the years and patient survival has improved, 20 percent to 30 percent of breast cancer patients still develop distant metastases within two years [4]. Furthermore, once it happens distant metastases with a five-year survival rate of only approximately 20 % [5]. However, the exact molecular mechanism underlying breast cancer metastasis is still incompletely understood. Therefore, novel carcinogenic pathways underlying breast cancer progression and metastases should be elucidated.

Dicer is a crucial RNAse III endonuclease in the cytoplasm and plays a vital role in miRNA processing and siRNA maturation [6]. Alterations in Dicer expression levels and activity are involved in tumor progression through an overall silencing in miRNA expression [7]. In addition, the downregulation of Dicer expression plays a significant role in the growth, migration, and invasion of cancer cells [8–10]. Chen et al. reported that Dicer reduced cell migratory and invasive abilities by inhibiting matrix metalloproteinase-2 (MMP-2) expression in kidney cancer [11]. Moreover, a recent study showed that the inhibition of Dicer confers metastasis in breast cancer [12], promoted the cancer stemness phenotype and enhanced metastatic potential in colorectal cancer. Clinical data have also demonstrated the correlation of decreased Dicer expression with distant metastasis and poor survival outcomes in liver cancer [10], ovarian cancer [13], cervical cancer [9], and breast cancer [14, 15]. Nevertheless, whether Dicer regulates tumor metastasis and regulatory mechanisms of breast cancer remains unclear. Therefore, identifying novel molecules and new therapeutic targets in metastatic breast cancer is warranted.

Cancer stem cells (CSCs) undergo self-renewal and exhibit resistance to apoptosis and drugs and have high metastatic potential [16, 17]. Among them, large-scale reports showed that breast CSCs are considered the principal cause of cancer progression, metastasis, drug resistance, and recurrence in breast cancer [18, 19]. However, most of the current therapeutic modalities for distant metastases in breast cancer are not effective against CSCs. Recently, some reports demonstrate that miRNAs play essential roles in regulating cancer cell migration, invasion, and stemness. In prostate cancer, the silencing of miR-218 promoted cell migration and cancer stem cell properties [20]. Moreover, the inhibition of miR-135b suppressed cell migration, invasion, and stemness in pancreatic cancer cells through the AKT/mTOR pathway [21]. Loss of Dicer expression enhanced the migratory and invasive abilities and stemness of endometrial carcinoma by reducing the expression of the let-7 family of miRNAs [22]. However, few studies have examined whether Dicer-regulated miRNAs affect cell migration, invasion, and CSCs properties in breast cancer.

In this study, we elucidated the mechanisms through which Dicer enhances the cell migration and invasion of breast cancer cells. We determined that the protein expression level of Dicer significantly decreased in highly invasive breast cancer cells (MCF-7/I-3 and MDA-MB-231/I-3). Moreover, the results of The Cancer Genome Atlas (TCGA) database analysis showed that Dicer expression was inversely associated with the breast cancer stage compared with normal breast, and a lower Dicer expression level was correlated with poor overall survival in patients with breast cancer. Knockdown of Dicer expression increased the cell migration, invasion and CSCs properties of breast cancer cells, whereas the overexpression of Dicer reduced the cell migration, invasion and CSCs properties in breast cancer cells. More importantly, we further indicated that miR200b abolished the increase in the migration, invasion, and stemness of cancer cells caused by the knockdown of Dicer expression. Our findings imply that Dicer suppression contributes to the enhanced migration, invasion, and CSCs properties of breast cancer cells.

## RESULTS

### Dicer, but not other miRNA biogenesis enzyme, is markedly downregulated in highly invasive breast cancer cells

**Figure 1:**
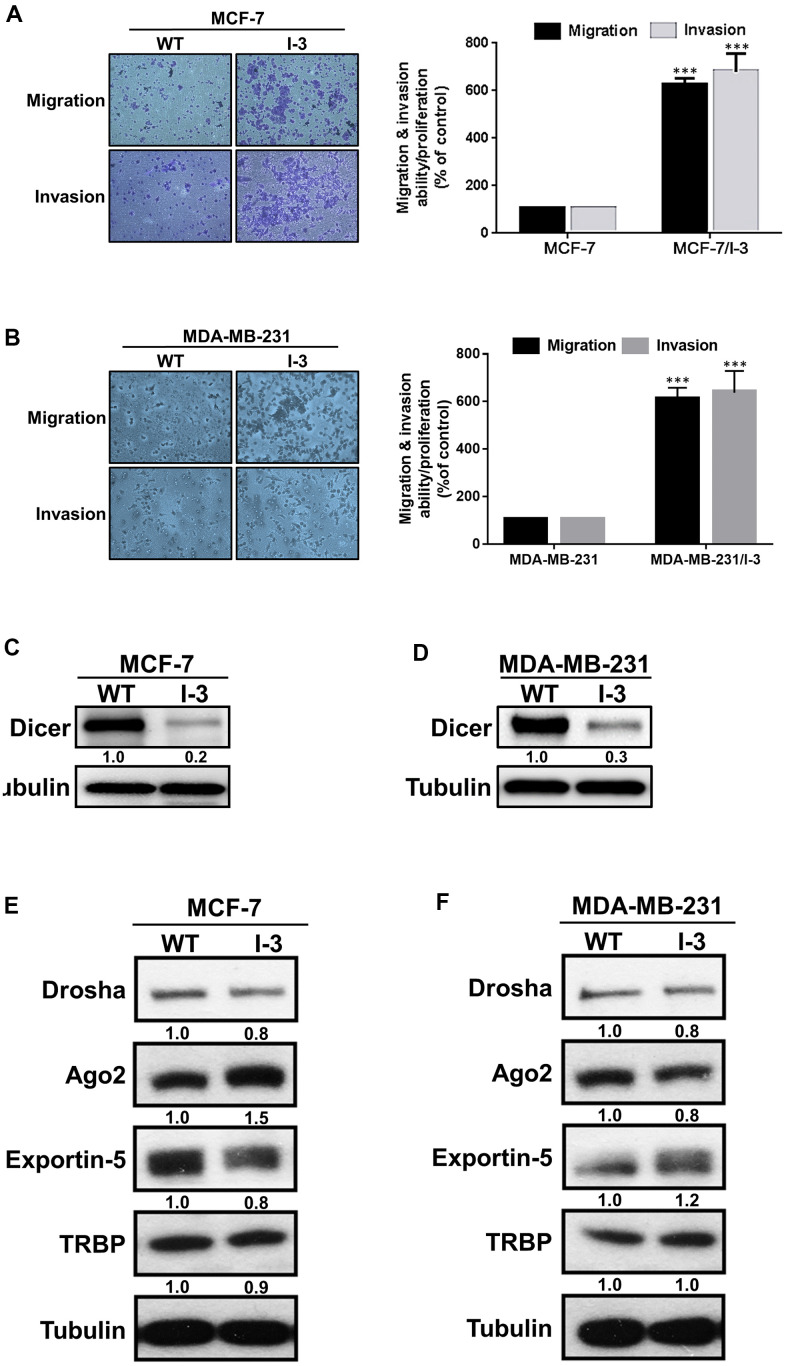
**Dicer is significantly downregulated in highly invasive breast cancer cells.** (**A**, **B**) A panel of highly invasive breast cancer (MCF-7/I-3 and MDA-MB-231/I-3) cells was established, and their migration and invasion abilities were evaluated by performing cell migration and invasion assays, respectively. (**C**, **D**) The protein expression levels of Dicer in highly invasive cells (MCF-7/I-3 and MDA-MB-231/I-3) and parental cells (MCF-7 and MDA-MB-231). (**E**, **F**) Analysis of miRNA biogenesis–related protein expression levels in highly invasive cells (MCF-7/I-3 and MDA-MB-231/I-3) cells compared with those in parental cells (MCF-7 and MDA-MB-231) through Western blotting. Data are presented as the mean ± standard error mean of three independent experiments. ***P < 0.001.

To examine the mechanisms underlying breast cancer metastasis, we established highly invasive cell lines (MCF-7/I-3 and MDA-MB-231/I-3) by performing the *in vitro* Matrigel-coated Transwell assays and determined the migratory and invasive abilities by conducting Transwell migration and invasion assays, respectively. Our data revealed that cell migratory and invasive abilities were markedly increased in the highly invasive cells compared with their parental cells. (Figure 1A, 1B). These results confirmed the successful establishment of the highly invasive cell lines.

Next, we examined the expression levels of miRNA biogenesis-related proteins in the parental and highly invasive cells and observed that Dicer expression was lower in the highly invasive cells (Figure 1C, 1D). Moreover, we examined the expression levels of other miRNA biogenesis–related proteins, namely Drosha, Ago2, exportin-5, and TAR-RNA binding protein (TRBP), in the highly invasive and parental cells. The expression levels of these miRNA machinery proteins did not significantly differ between the highly invasive and parental cells. (Figure 1E, 1F). The aforementioned results indicate that Dicer plays a crucial role in the migration, invasion, and metastasis of breast cancer.

### Dicer expression is correlated with patient survival

**Figure 2:**
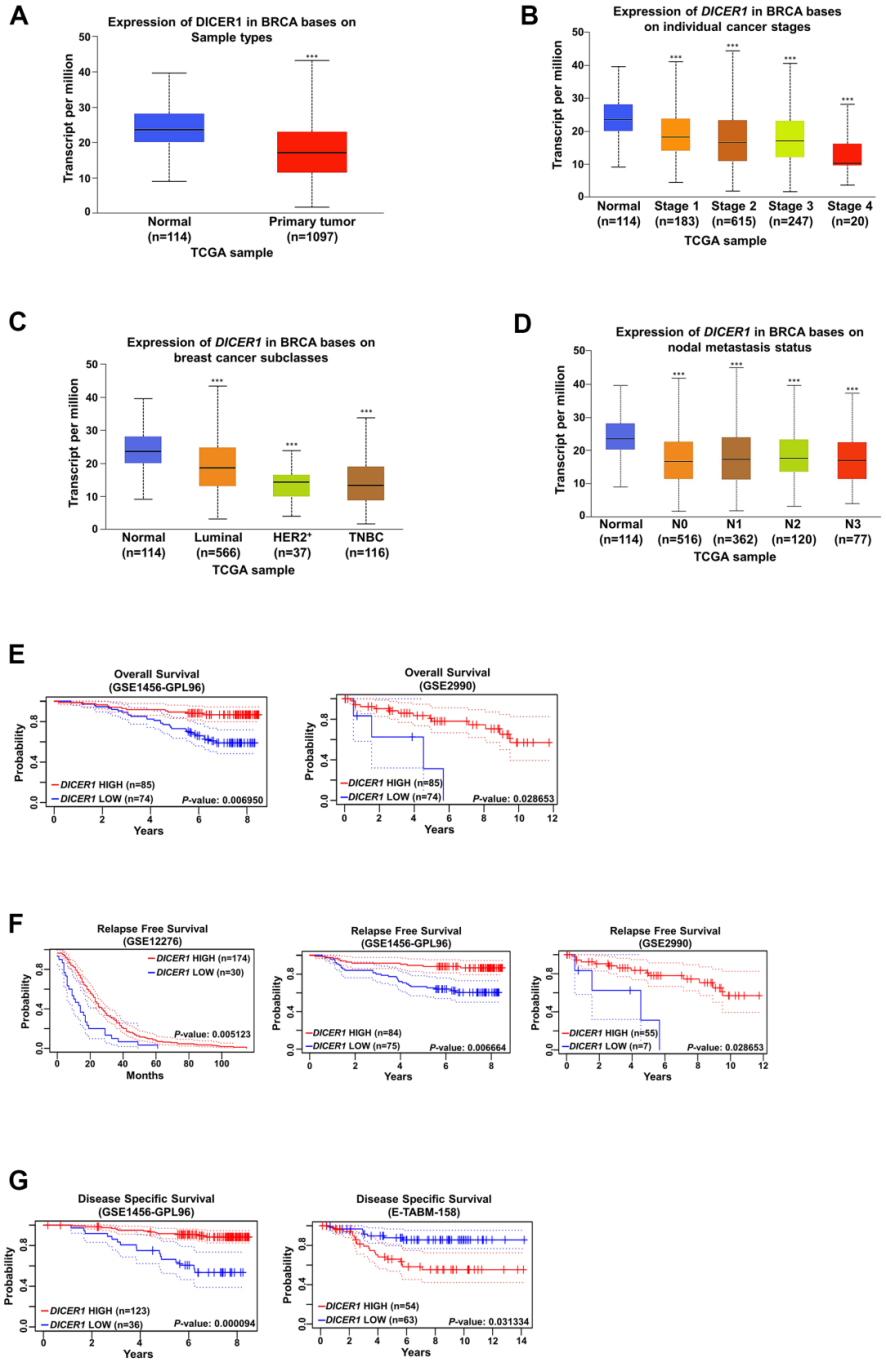
**Analysis of Dicer expression in breast cancer.** (**A**) Analysis of Dicer expression in the breast cancer tissue and normal breast tissue by using TCGA database generated from UALCAN (http://ualcan.path.uab.edu/index.html). Dicer expression was negatively correlated with different (**B**) breast cancer stages, (**C**) breast cancer subtypes, and (**D**) nodal metastasis status. The Kaplan–Meier plot shows the (**E**) overall survival curves, (**F**) relapse-free survival curves, and (**G**) disease-specific survival curves of patients with breast cancer with different Dicer expression levels. Data are presented as the mean ± standard error mean of three independent experiments. ***P < 0.001.

To determine Dicer expression in the patients with breast cancer, we investigated its relationship with the patients’ clinical parameters by using TCGA data from the UALCAN website (http://ualcan.path.uab.edu/). The results demonstrated that the Dicer expression levels was lower in breast cancer tissues than in normal breast tissues (Figure 2A). Furthermore, we analyzed Dicer expression in the patients with different clinical breast cancer stages. Dicer expression was markedly lower in breast cancer tissues at the different stages than in normal breast tissue (Figure 2A, 2B).

Because breast cancer is divided into distinct subtypes, we analyzed Dicer expression in different breast cancer subtypes and determined markedly lower Dicer expression in the luminal, human epidermal growth factor receptor 2-positive (HER^+^), and triple-negative breast cancer (TNBC) subtypes than in the normal breast tissue (Figure 2C). Moreover, we observed that low Dicer expression was significantly relevant to lymph node metastasis (Figure 2D). Furthermore, we evaluated the relevance of Dicer expression levels for breast cancer prognosis by using PrognoScan (http://www.prognoscan.org/). Low Dicer expression in breast cancer patients was markedly correlated with poor overall survival. (Figure 2E). Moreover, low Dicer expression was also correlated with poor relapse survival rate (Figure 2F) and disease-specific survival (Figure 2G). These results indicate that Dicer expression is associated with poor prognosis in breast cancer patients.

### Inhibition of Dicer expression promotes the migration, invasion, and CSCs properties

**Figure 3:**
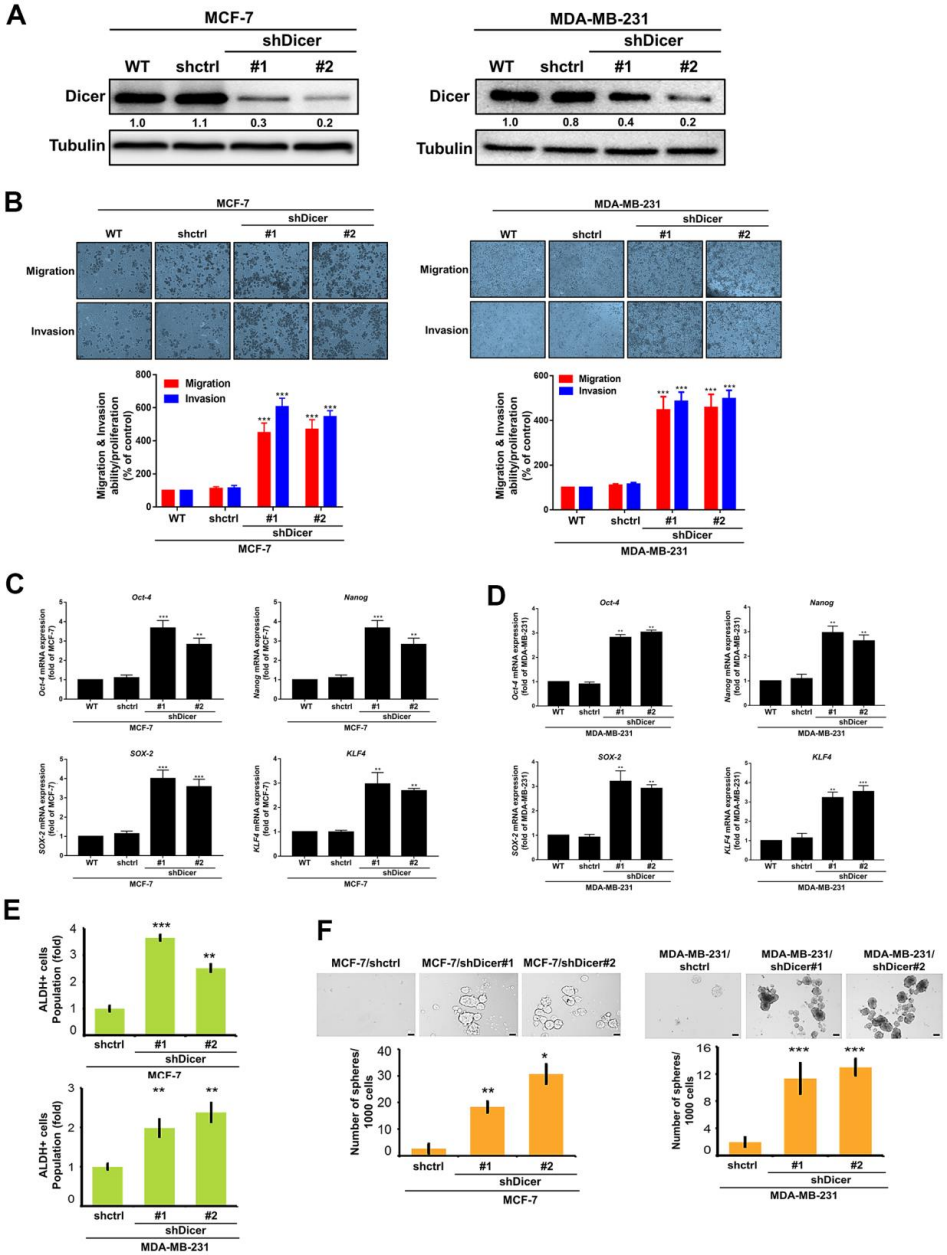
**Dicer silencing enhances the migration/invasion, and CSCs properties of breast cancer cells.** (**A**) The stable Dicer-silenced MCF-7 and MDA-MB-231 cells were analyzed through Western blotting. (**B**) The migration and invasion of the stable knockdown breast cancer cells were evaluated by performing cell migration and invasion assays, respectively. (**C**, **D**) qRT-PCR analysis of *Oct-4, Nanog, SOX-2, and KLF4* in Dicer-knockdown MCF-7 and MDA-MB-231 cells. (**E**) Analysis of ALDH activity through flow cytometry to determine the subpopulation of CSCs properties. (**F**) Sphere formation assay to evaluate self-renewal and differentiation at the single-cell level *in vitro* in the present cells. Data are presented as the mean ± standard error mean of three independent experiments. *P < 0.05, **P < 0.01, ***P < 0.001.

We noted significantly lower Dicer expression in the highly invasive cells. Moreover, some studies have reported that Dicer plays an important role in regulating tumor progression in different types of cancer [8, 23]. Thus, in the present study, to investigate whether Dicer affects the migration, invasion, and CSCs properties of breast cancer cells, we established stable Dicer-silenced (MCF-7/shDicer and MDA-MB-231/shDicer) cells, respectively (Figure 3A). Moreover, we further evaluated the effects of Dicer on the cell migratory/invasive abilities and found that Dicer silencing enhanced both cell migration and invasion (Figure 3B). In addition, cancer stemness is a primary driver of cancer recurrence and metastasis [24–26]. Therefore, we surmised that Dicer participates in regulating the CSCs properties of breast cancer cells. The results of reverse transcription-quantitative polymerase chain reaction (qRT-PCR) demonstrated that the knockdown of Dicer significantly increased the expression of the stemness-related transcriptional factor markers *Oct-4, Nanog, SOX-2, and KLF4* (Figure 3C, 3D). Consistently, the results of the aldehyde dehydrogenase 1 (ALDH1) activity assay revealed that ALDH1 activity was higher in the shDicer cells than in the knockdown control (shCtrl) cells (Figure 3E). Furthermore, we examined the self-renewal ability of CSCs and observed that Dicer knockdown increased the diameter and quantity of mammospheres (Figure 3F). These data indicate that the knockdown of Dicer expression can promote the cell migratory/invasive abilities and enhanced CSCs properties in breast cancer cells.

### Dicer overexpression reduces migration, invasion, and CSCs properties

**Figure 4:**
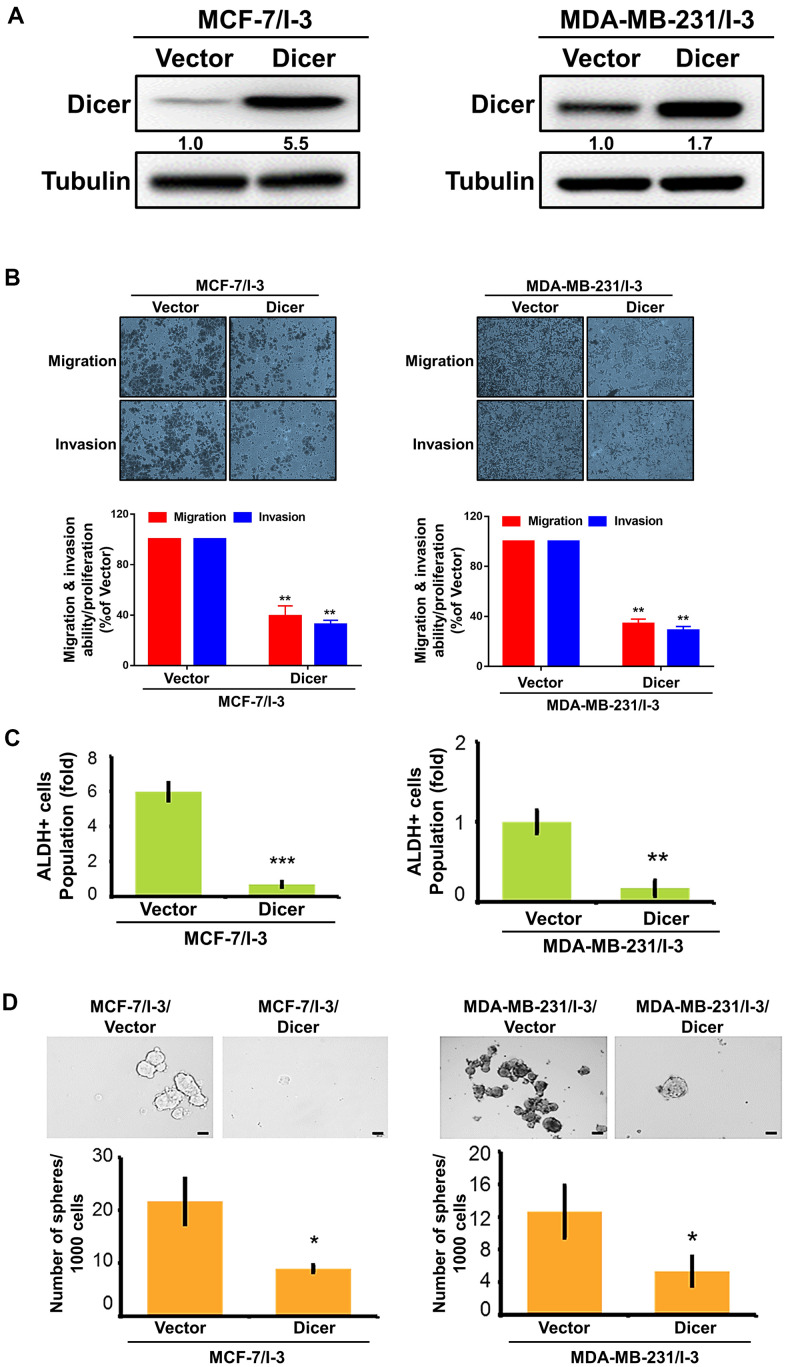
**Overexpression of Dicer reduced enhances the migration, invasion, and stemness of breast cancer cells.** (**A**) Western blot analysis of Dicer overexpression in highly invasive (MCF-7/I-3 and MDA-MB-231/I-3) cells. (**B**) The migration and invasion of the indicated cells were examined using the cell migration and invasion assays, respectively. (**C**) Flow cytometry of ALDH activity in Dicer-overexpressing breast cancer cells. (**D**) Self-renewal ability was examined in Dicer-overexpressing breast cancer cells by using the sphere formation assay. Data are presented as the mean ± SEM of three independent experiments. *P < 0.05, **P < 0.01, ***P < 0.001.

To investigate whether Dicer affects the cell migratory/invasive abilities and CSCs properties of the breast cancer, we transfected Dicer-overexpressing plasmids into the highly invasive cells and determined the overexpression efficiency of Dicer through a Western blot analysis (Figure 4A). The overexpression of Dicer enhances the cell migratory/invasive abilities of the highly invasive breast cancer cells, whereas silencing of Dicer markedly reduced the cell migratory/invasive abilities of breast cancer (Figure 4B). Moreover, Dicer overexpression significantly reduced ALDH activity (Figure 4C). We determined the self-renewal ability of the breast cancer cells through the sphere formation assay, and the results revealed that the overexpression of Dicer inhibited sphere formation (Figure 4D). These results indicated that the overexpression of Dicer suppresses the migration, invasion, and CSCs properties of breast cancer cells.

### miR-200b is involved in Dicer-mediated migration, invasion, and CSCs properties

**Figure 5:**
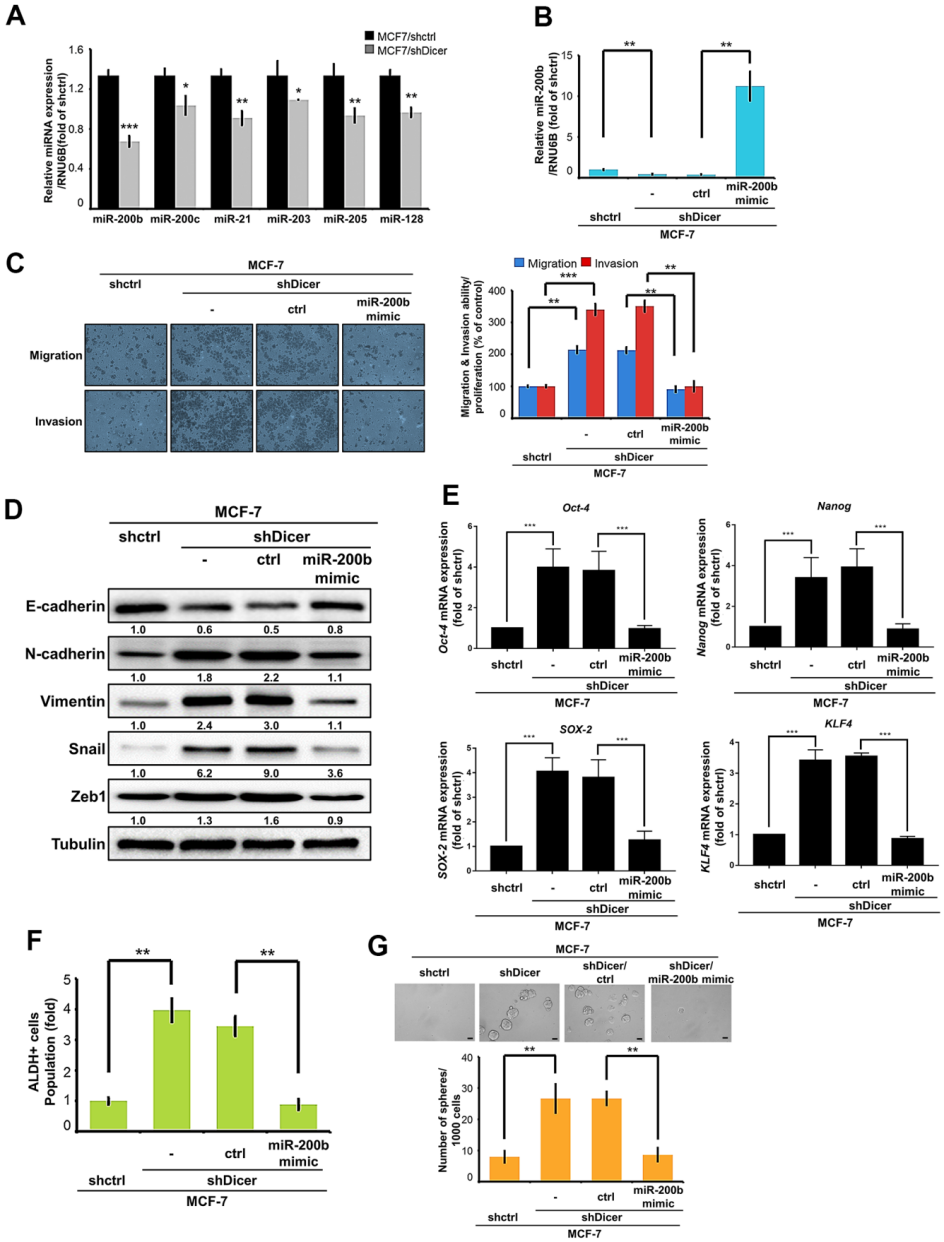
**miR-200b is involved in Dicer-mediated cell migration/invasion, and CSCs properties of breast cancer cells.** (**A**) Analysis of miR-200b, miR-200c, miR-21, miR-203, miR-205, and miR-128 in MCF-7/shDicer cells compared with MCF-7 cells through quantitative real-time polymerase chain reaction (qRT-PCR). (**B**) qRT-PCR analysis was performed to detect miR-200b levels in the MCF-7/shDicer cells transfected with a miR-200b mimic. (**C**) The migration and invasion of the indicated cells were examined using the cell migration and invasion assays, respectively. (**D**) The protein expression levels of E-cadherin, N-cadherin, Vimentin, Snail, and Zeb1 were analyzed through Western blot. (**E**) The expression levels of *Oct-4, Nanog, SOX-2, and KLF4* mRNA were analyzed through qRT-PCR. (**F**) Analysis of ALDH activity to determine the subpopulation of CSCs properties of the present cells. (**G**) Sphere formation was examined to measure the self-renewal ability of MCF-7 cells with Dicer knockdown and miR-200b overexpression. Data are presented as the mean ± standard error mean of three independent experiments. *P < 0.05, **P < 0.01, ***P < 0.001.

Dicer is a major enzyme in the miRNA biogenesis machinery and is crucial for miRNA processing and maturation. Accumulating evidence demonstrates that several miRNAs (miR-200b, miR-200c, miR-21, miR-203, miR-205, and miR-128) may be correlated with the epithelial mesenchymal transition (EMT) and CSCs properties in breast cancer cells [27]. Thus, we focused on these relevant miRNAs and determined whether their expression affects the EMT and cancer stemness of the cancer cells. The results of real-time RT-PCR revealed that the levels of these candidate miRNAs decreased in the MCF-7/shDicer cells. Among them, miR-200b appeared as the most significant difference in MCF-7/shDicer cells and MCF-7/shCtrl cells (Figure 5A). By contrast, the overexpression of Dicer in the MCF-7/I-3 cells significantly increased miR-200b expression (Supplementary Figure 1A). Moreover, we also found that miR-200b was downregulated in the highly invasive breast cancer cells (MCF-7/I-3 and MDA-MB-231/I3) compared with the parental cells (Supplementary Figure 1B). Increasing evidence has implicated that miR-200b may participate in tumor progression and stemness regulation [28, 29]. Thus, we hypothesized that Dicer enhances the cell migratory/invasive abilities, and CSCs properties of breast cancer cells through miR-200b. To confirm this hypothesis, we transfected a miR-200b mimic into the MCF-7/shDicer cells and confirmed its transfection efficiency through RT-qPCR (Figure 5B). Next, we investigated whether the overexpression of miR-200b abolished Dicer knockdown induced cell migratory/invasive abilities. Our results showed that transfecting a miR-200b mimic into the MCF-7/shDicer cells successfully inhibited Dicer knockdown induced cell migratory/invasive abilities (Figure 5C). Consistently, the inhibition of miR-200b expression resulted in the recovery of the cell migratory/invasive abilities in MCF-7/I-3/Dicer cells (Supplementary Figure 1C). In addition, we investigated whether miR-200b affects the expression of EMT markers in the MCF-7/shDicer cells and observed that the transfection with a miR-200b mimic into the MCF-7/shDicer cells reduced N-cadherin, Vimentin, snail, and Zeb-1 expression levels and increased E-cadherin expression level (Figure 5D). Moreover, we also examined whether Dicer inhibition promoted cancer stemness properties through miR-200b. We performed qRT-PCR to analyzed the expression of cancer stemness markers and found that the overexpression of miR-200b in the MCF-7/shDicer cells reduced the expression of the stemness markers *Oct-4*, *Nanog*, *SOX-2*, and *KLF4* (Figure 5E). Consistent with the aforementioned findings, the inhibition of miR-200b restored the expression of the cancer stemness markers suppressed by the overexpression of Dicer (Supplementary Figure 1D). Furthermore, we examined whether Dicer inhibition promotes cancer stemness properties through miR-200b by performing ALDH activity assay. The overexpression of miR-200b significantly decreased ALDH activity in the MCF-7/shDicer cells (Figure 5F). Notably, we further evaluated the self-renewal ability of the CSCs by performing a sphere formation assay; the results revealed that the inhibition of Dicer expression increased sphere diameter and quantity. By contrast, the overexpression of miR-200b in the MCF-7/shDicer cells reduced sphere diameter and quantity (Figure 5G). These results suggest that Dicer mediates the cell migratory/invasive abilities and cancer stemness properties of breast cancer cells by regulating miR-200b.

## DISCUSSION

Breast cancer is the most frequently diagnosed primary malignancy and the leading cause of mortality in women in the worldwide [30]. Although breast cancer treatment has significantly improved and prolonged the patients’ survival, the prognosis of patients with breast cancer remains poor due to being prone to distant metastasis [4]. Unfortunately, the pathobiological features of and the exact mechanism underlying distant metastasis of breast cancer remain incompletely understood and warrant further research.

The components of the miRNA biogenesis machinery have been implicated in cancer progression, metastasis, and drug resistance [7, 31, 32]. Among them, Dicer is crucial for miRNA processing and maturation. Large-scale studies have indicated that alterations in the expression levels or activity of Dicer may personate as oncogenes or tumor suppressors in cancers and may result in poor prognosis in many types of cancer [33–35]. For instance, Dicer inhibition increased renal cell carcinoma metastasis [11], and enhanced the cell migration/invasion of cervical cancer cells [9]. In addition, the inhibition of Dicer expression enhanced the mesenchymal phenotype and promoted bladder cancer cell invasion by suppressing MMP-2 expression [36]. Impaired Dicer expression reduced the cisplatin sensitivity, cell proliferation and cell migration of ovarian cancer cells [37]. Moreover, the overexpression of Dicer decreased the cell proliferation, migration and invasion of hepatocellular carcinoma (HCC) cells through the vascular endothelial growth factor signaling pathway [38]. On the contrary, Dicer knockdown increased drug sensitivity in gemcitabine-resistant pancreatic cancer cells [23]. In the current study, we established highly invasive cell lines to investigate the molecular mechanisms underlying cell migration and invasion. Our results revealed that the highly invasive cells exhibited lower Dicer expression than did the parental cells. Moreover, the inhibition of Dicer expression was correlated with significantly increased cell migratory/invasive abilities. Clinical studies have reported that lower Dicer expression was significantly correlated with poor prognosis in multiple cancers, such as ovarian cancer [13], glioblastoma [9], renal cell cancer [39], liver cancer [40], and lung cancer [41]. These findings are similar to those of our TCGA dataset analysis. Dicer expression was significantly lower in the breast cancer tissue then in the normal breast tissue. Furthermore, the Dicer expression level significantly decreased with the progression of the tumor stage and nodal metastasis status in the patients with breast cancer. Moreover, low Dicer expression led to worse overall survival rates, recurrence-free survival rates and disease-specific survival rates. These results indicate that Dicer expression has an inextricable relationship with cell migratory/invasive abilities and leads to poor prognosis in breast cancer patients.

Cancer stemness properties include self-renewal and multipotent differentiation characteristics, which are perceived as sources of tumorigenesis, metastasis, and drug resistance [26, 42, 43]. For instance, some studies have reported that TBL1XR1 promotes CSCs properties and metastasis in gastric cancer [44]. In addition, the inhibition of UBQLN1 reduced the invasion and cancer stemness of breast cancer [45]. Moreover, the inhibition of Dicer expression enhanced the CSCs properties and metastasis in colon cancer cells [46]. A decreased Dicer level increased the expression of the cancer stemness markers CD44, Aldh1, OCT, and Nanog and promoted the self-renewal of endometrial carcinoma cells [22]. Repression of Dicer expression increased the number of HCC cells with CD133-positive and CD44-positive phenotypes [47]. However, the correlation of Dicer expression and cancer stemness properties in breast cancer remains unclear. Therefore, we speculate that the decreased expression of Dicer promotes breast cancer metastasis by enhancing cancer stemness properties. Consistent with this speculation, our findings demonstrated that the inhibition of Dicer expression increased ALDH activity and self-renewal ability. Moreover, the overexpression of Dicer in the highly invasive breast cancer cells significantly reduced ALDH activity and self-renewal ability. The current results indicate that Dicer is a novel suppressor gene that regulates the CSCs properties of breast cancer cells.

**Figure 6:**
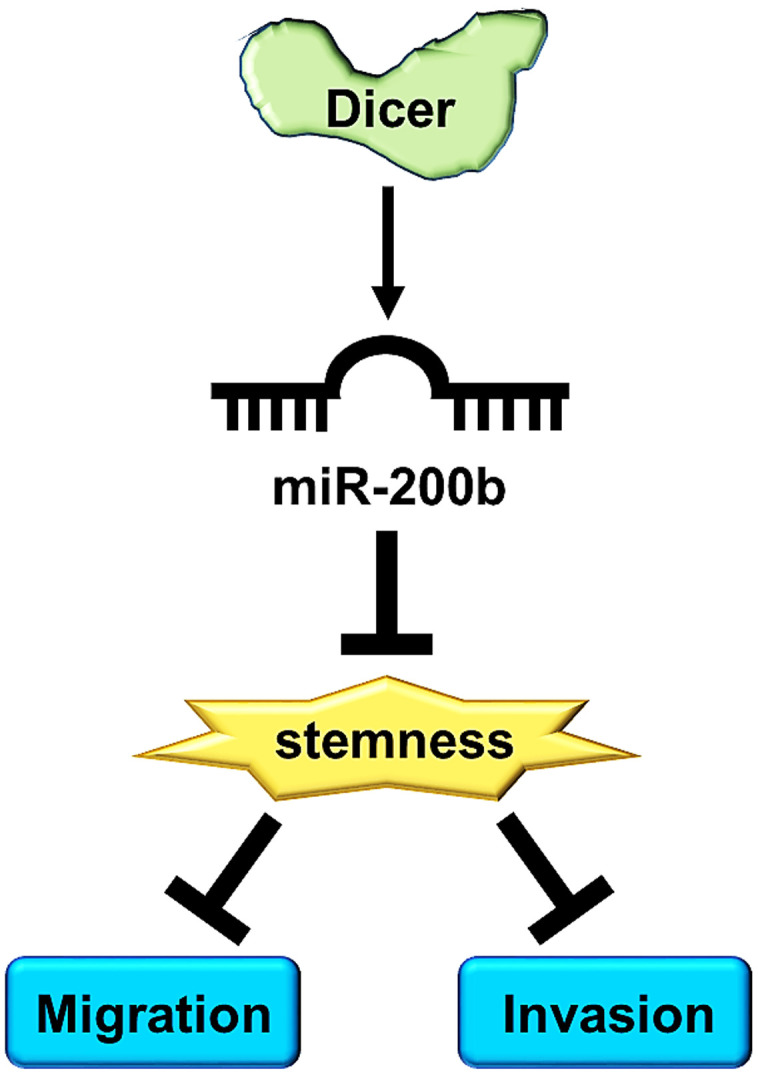
A schematic diagram illustrates that Dicer mediates the cell migration/invasion, and CSCs properties of breast cancer through miR-200b regulation.

Dicer is an essential miRNA biogenesis enzyme, crucial for the processing of pre-miRNAs into mature miRNA. In addition, miRNAs regulate cancer metastasis and cancer stemness in different cancer types [48]. For instance, miR-3065-3p promotes the cell migration, invasion and enhances cancer stemness in colorectal cancer [49], and miR-590-5p was reported to decrease stemness and metastasis in breast cancer [50]. Thus, these studies suggest that miRNAs may play an essential role in the fight against cancer stemness and cancer metastasis. Recent studies have reported that miR-200b is frequently downregulated in various cancers and exerts prominent effects on cell migration, invasion, and drug resistance [51]. Inhibition of miR-200b expression promoted EMT and invasion in colon cancer cells and esophageal squamous cell carcinoma (ESCC) cells [46]. Moreover, MALAT1 enhanced docetaxel resistance in breast cancer cells by reducing miR-200b expression [52]. 53BP1 suppressed the cell migration and invasion of breast cancer cells by upregulating miR-200b expression [53]. Our results indicated that miR-200b expression was lower expressed in the highly invasive cells and MCF-7/sh Dicer cells. More importantly, we also demonstrated that miR-200b abolished Dicer knockdown induced the cell migratory/invasive abilities and cancer stemness of breast cancer cells (Figure 6). In addition, we examined the relationship between Dicer and miR-200b by using online clinical databases. However, we did not determine the association between Dicer and miR-200b in breast cancer clinical databases; this is a limitation of the present study. Therefore, future studies should examine the correlation between Dicer and miR-200b and the regulatory mechanism of Dicer-mediated miR-200b inhibition of breast cancer stemness and metastasis.

Our current findings revealed the mechanism through which Dicer regulate metastasis and CSCs properties regulation in invasive breast cancer cells. In conclusion, Dicer can be a novel diagnostic marker and potential therapeutic target for the treatment of metastatic breast cancer.

## MATERIALS AND METHODS

### Cell culture and highly invasive cell line establishment

The human breast cancer cell lines MCF-7 and MDA-MB-231 were purchased from the Bioresource Collection and Research Center (BCRC, Hsinchu, Taiwan). The MCF-7 cells were cultured in RPMI 1640 medium (Gibco, New York, NY, USA), and the MDA-MB-231 cells were preserved in Dulbecco’s modified Eagle’s medium (DMEM)/F12 medium (Gibco); both the media were supplemented with 10% fetal bovine serum (Gibco) and 1% penicillin–streptomycin solution (Gibco).

To establish the breast cancer cell lines with high invasive ability, we used the BioCoat Matrigel Invasion Chamber (Corning, NY, USA). This chamber consists of an upper chamber and a lower chamber, with the upper chamber having 8-μm holes at the bottom and being precoated with extracellular matrix proteins. The MCF-7 and MDA-MB-231 cells invaded the lower external portion of their respective chambers through the Matrigel. The breast cancer cells were seeded at a density of 2.5 × 10^4^ in the upper chamber and incubated for 24 h at 37° C in 5% CO2. When a sufficient number of invasive cells were produced, we initiated a second round of invasive cell selection. In brief, the increased number of cells was inoculated into the upper compartment of a new chamber, and this experiment was repeated three times. The final cell lines were considered to be highly invasive (I-3), and the original cell lines were used as the parental cells in subsequent experiments.

### Transwell migration and invasion assay

The Transwell migration assay was performed using uncoated Transwell chambers with 8-μm pores (BD Biosciences). The cells were plated with serum-free medium in the upper chamber, whereas complete medium was added to the lower chamber. After 12 h, by using cotton swabs, we removed the cells that had not migrated from the upper chamber. Next, we used methanol to fix the cells that migrated to the lower chamber. Finally, the migrated cells were stained with crystal violet (Sigma-Aldrich, St. Louis, MO, USA). The Transwell invasion assay was performed using the BioCoat Matrigel Invasion Chambers (Corning). The cells were plated in the top chamber onto the Matrigel-coated membrane, and the detailed procedure has been described previously. All the images of the migrated and invaded cells were obtained under a microscope at 100× magnification.

### Western blotting

The cells were lysed in RIPA lysis buffer containing a protease inhibitor cocktail at −20° C for 2 h and centrifuged at 13,300 rpm for 30 min. Next, the supernatants (i.e., protein lysates) were collected, and an equal quantity of protein from the lysates was heated at 100° C for 10 min in sample buffer. An equal quantity of protein was resuspended in gel sample buffer for sodium dodecyl sulfate–polyacrylamide gel electrophoresis, and the separated protein was transferred onto the polyvinylidene fluoride (PVDF) membrane (Millipore, Burlington, MA, USA) by using the all-wet method (Bio-Rad). After blocking, we incubated the PVDF membrane with specific primary antibodies. Subsequently, the membrane was incubated with secondary antibodies. Finally, the blot with immunoreactive proteins was examined using a chemiluminescence detection system (Millipore-Crescendo, Burlington, MA, USA). The primary antibodies against the following proteins were used for Western blotting: Dicer (ab14601, Abcam, Cambridge, UK), Drosha (ab12286, Abcam), AGO2 (ab32381, Abcam), TRBP (15753-1-AP, Proteintech, Chicago, IL, USA), exportin-5 (12565, Cell Signaling, Danvers, MA, USA), E-cadherin (3195, Cell Signaling), N-cadherin (ab18203, Abcam), vimentin (ab92547, Abcam), Snail (A5243, Abclonal, Woburn, MA, USA), Zeb-1 (3396, Cell Signaling), and α-tubulin (sc-8035, Santa Cruz Biotechnology, Dallas, TX, USA). The Western blotting data were quantified using ImageJ software and normalized with the tubulin expression level.

### Dicer plasmid transfection

To induce Dicer expression in the cells, the cDNA of Dicer genes was cloned into pcDNA6 (Invitrogen, Waltham, MA, USA) and transfected into the cells by using the TOOLSFect transfection reagent (Biotools Co., Ltd., Taipei, Taiwan). The transfected cells were selected using blasticidin and diluted to obtain stable clones. The lentiviral Dicer short-hairpin RNA (shDicer) and the negative control (shCtrl) constructs were created using the same vector system, which was purchased from National RNAi Core Facility, Academica Sinica, Taipei. The lentivirus stock was developed in accordance with the manufacturer’s protocol. We used 2 μg/mL of puromycin for the selection of the stable knockdown cells 48 h after lentivirus infection. Knockdown efficiency was determined through Western blotting and RT-qPCR. All plasmids were extracted using the Tools Plasmid Mini Kit (BIOTOOLS Co., Ltd., Taipei, Taiwan).

### qRT-PCR

Total RNA was isolated using the RNeasy Mini Kit (Qiagen, Hilden, Germany) and was reverse transcribed into cDNA by using the Quantitect Reverse Transcription Kit (Qiagen) in accordance with the manufacturer’s instructions. The target genes were quantified using qPCRBIO SyGreen 1-Step Detect/1-Step Go (PRO, Tech). GAPDH was used as the reference gene. To isolate miRNAs, total RNA was isolated using the miRNeasy Mini Kit (Qiagen) and was reverse transcribed using the miRNA Reverse Transcription Kit (Qiagen) in accordance with the manufacturer’s protocol. U6B small nuclear RNA was used as the internal control.

### ALDH activity assay

ALDH activity was analyzed using the ALDEFLUOR kit (Stem Cell, France) in accordance with the manufacturer’s instructions. In brief, 1 × 106 cells were resuspended in 1 mL of ALDEFLUOR buffer. The ALDH substrate of BAAA was briefly mixed with the cells. Subsequently, the ALDH inhibitor diethylaminobenzaldehyde was added to the suspended cells; this mixture was treated as the negative control. We allowed the reaction to occur at 37° C for 40 min in both the tubes. Next, the cells were washed and suspended in the ALDEFLUOR assay buffer containing 1 μg/mL 4’,6-diamidino-2-phenylindole (DAPI) (Life Technologies, Switzerland), and the suspension was then analyzed through flow cytometry. The cells identified to have high ALDH activity were isolated.

### miRNA transfection

A miR-200b mimic and inhibitor were used to overexpress and knockdown miRNA in the different breast cancer cells, respectively. The cells were seeded into a 24-well plate and transfected with 5 nM mimic and 50 nM RNAiMAX Transfection Reagent (ThermoFisher Scientific, Carlsbad, CA, USA). After 24 h, fresh medium was added to the cells, and the culture was continued under normal conditions for subsequent experiments.

### Sphere formation assay

The cells were separated into single cells by using trypsin–ethylenediaminetetraacetic acid (EDTA), followed by two washes with phosphate-buffered saline to remove trypsin–EDTA. The cells at a density of 3000 cells/mL were resuspended in serum-free DMEM/F-12 medium supplemented with B-27 (Invitrogen), 20 ng/mL epidermal growth factor, and 10 ng/mL basic fibroblast growth factor (Invitrogen) in ultra-low attachment 6-well plates (Corning Inc.) for 7–14 days and were then imaged under an optical microscope.

### Statistical analysis

All statistical data are presented as mean ± standard deviation from three independent experiments. Statistical data were analyzed using GraphPad Prism (version 5.0 or 7.0; GraphPad, San Diego, CA, USA) by using two-tailed Student’s paired t-test and one-way analysis of variance to determine whether the results of the comparisons among groups were statistically significant. Statistical analysis was performed before data normalization, and a P value of <0.05 was considered statistically significant.

## Supplementary Material

Supplementary Figure 1
