# Supplementary material for: Dicer-mediated miR-200b expression contributes to cell migratory/invasive abilities and cancer stem cells properties of breast cancer cells
Source: Aging (Albany NY). 2022 Aug 8;14(16):6520–36. doi: 10.18632/aging.204205 (PMC9467414; doi:10.18632/aging.204205)
Supplement: Supplementary Figure 1 [file aging-14-204205-s001.pdf]

SUPPLEMENTARY FIGURE

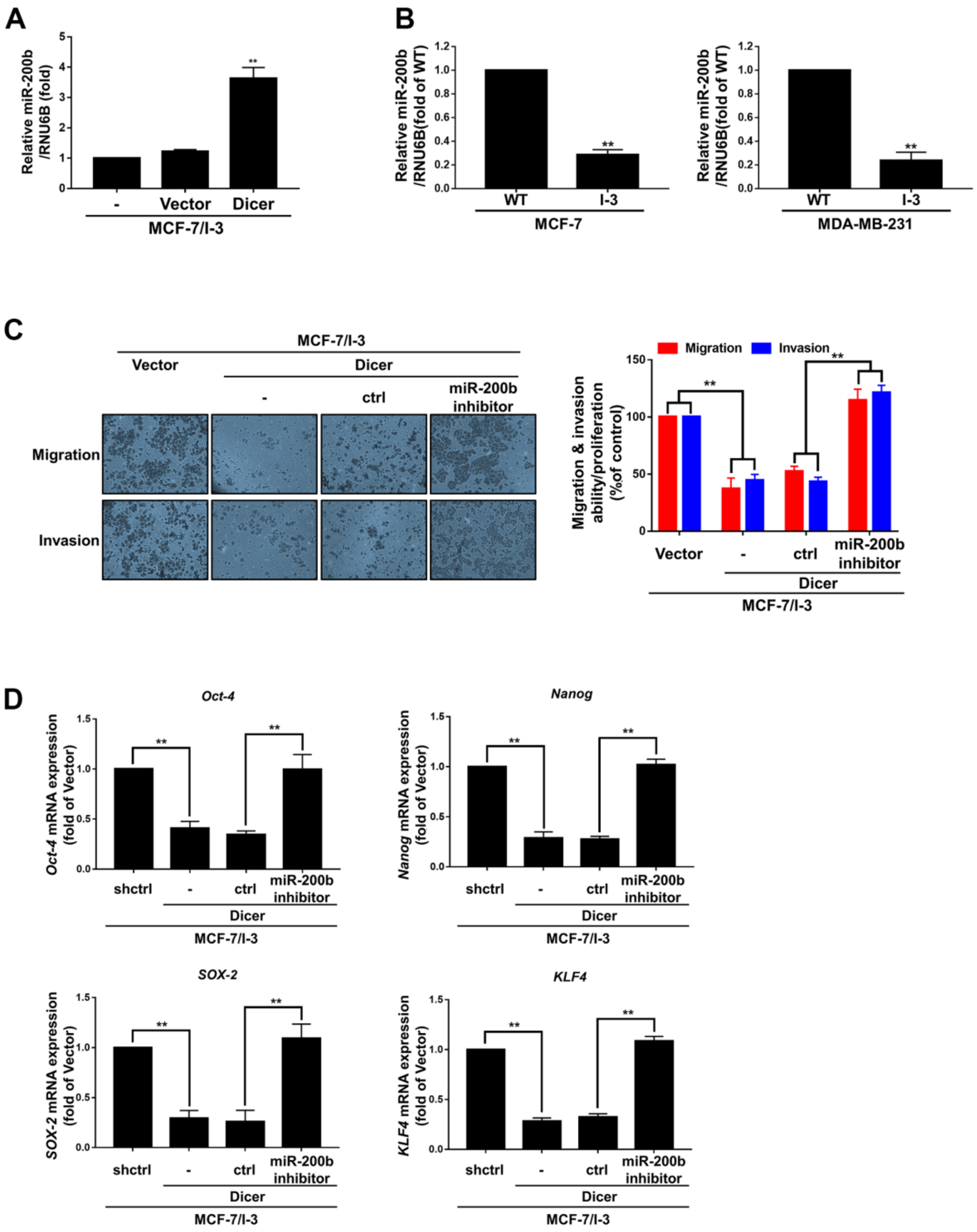

Supplementary Figure 1. Dicer-mediated migration/invasion and CSCs properties of breast cancer cells are regulated by miR-200b. (**A**) Analysis of miR-200b expression in Dicer-overexpressing MCF-7/I-3 cells through qRT-PCR. (**B**) Analysis of miR-200b expression in indicated cells. (**C**) The Transwell assay was performed to measure the migration and invasion of the indicated cells. (**D**) Expression of *Oct-4, Nanog, SOX-2, and KLF4* mRNA in the indicated cells was analyzed through qRT-PCR. Data are presented as the mean ± standard error mean of three independent experiments. **P < 0.01.
